# Supplementary figures and images for: Local risk perception enhances epidemic control
Source: PLoS One. 2019 Dec 3;14(12):e0225576. doi: 10.1371/journal.pone.0225576 (PMC6890219; doi:10.1371/journal.pone.0225576)

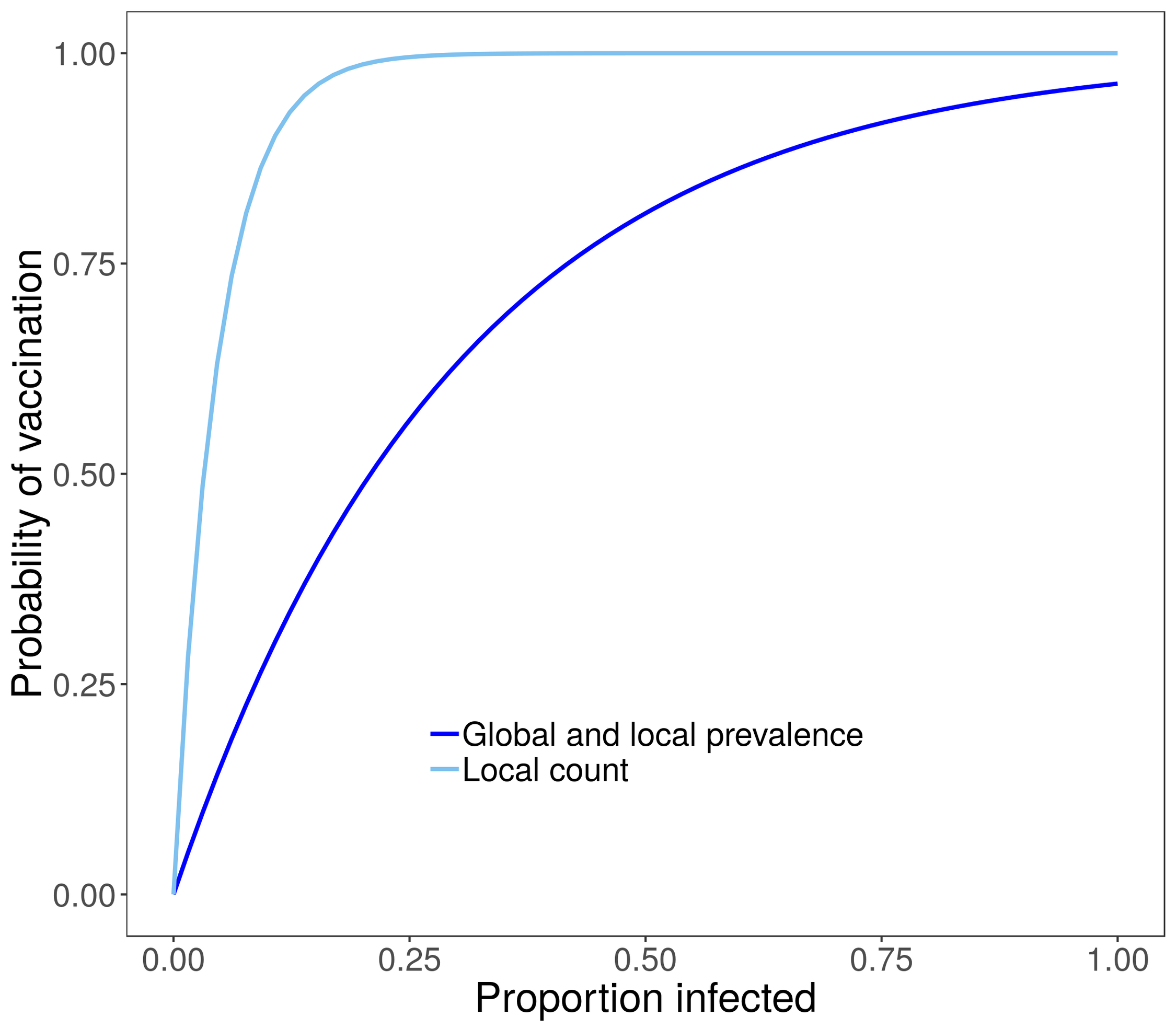

Supplement: S1 Fig — Assuming R0 = 5, an individual with k = 65 in our exponential network, we plot the probability of vaccination versus the proportion infected, which indicates either the fraction of neighbors infected (local prevalence and local count) or the overall fraction of the population infected (global prevalence). The behavior of the strategies that use prevalence is similar (blue); however, during the curse of a simulation, they have different values. (TIF) [file pone.0225576.s001.tif]

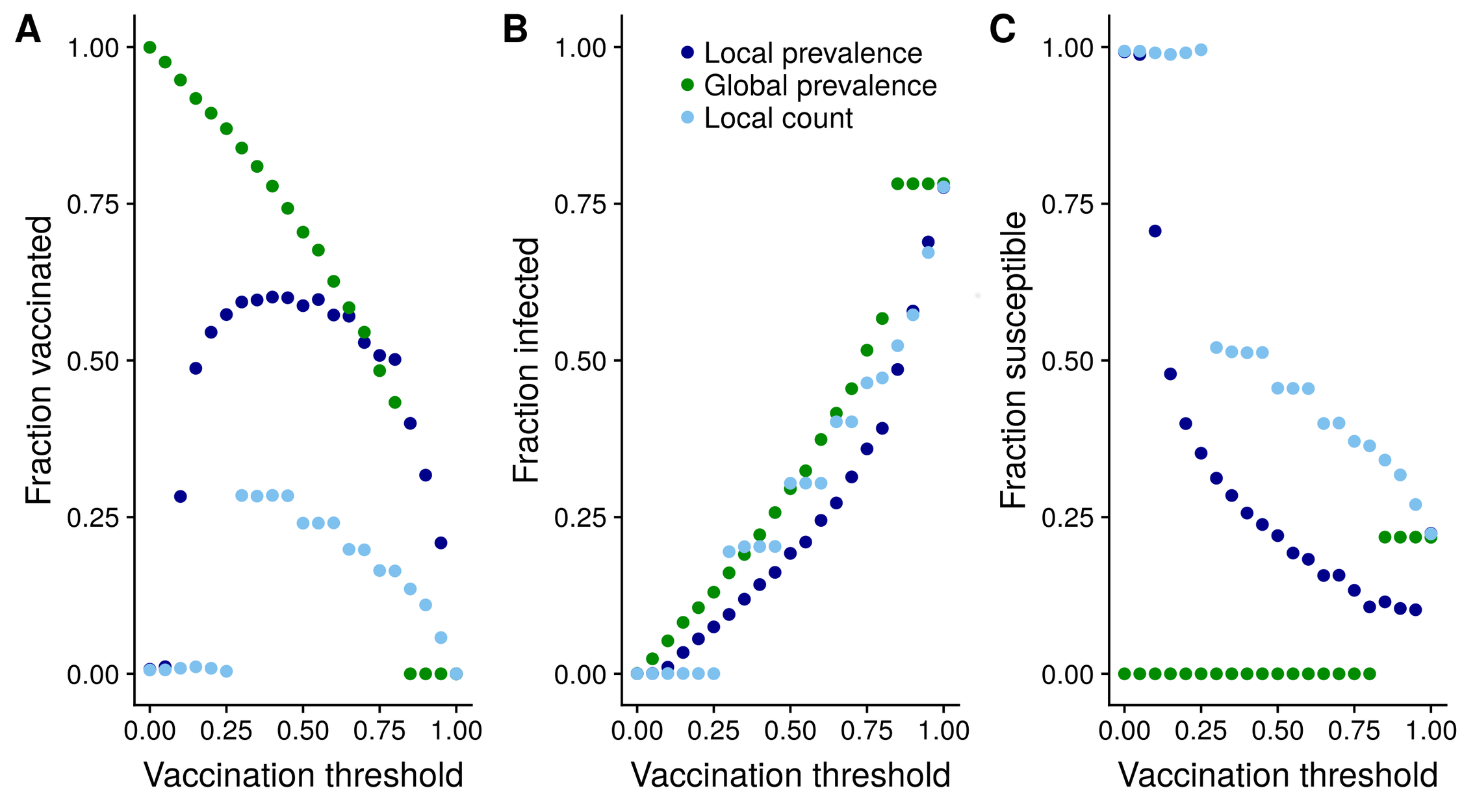

Supplement: S2 Fig — Individuals determine their infection risk according to the original local count, local prevalence or global prevalence equations. However, they vaccinate if and when their perceived risk crosses a specified vaccination threshold, rather than vaccinating probabilistically according to risk. This decision threshold (x-axes) impacts the proportion of the population that (A) vaccinates (and are not infected), (B) becomes infected (with or without vaccination), and (C) remains susceptible, under the three different decision models. Y-axes values are means across 500 stochastic simulations, assuming R0 = 5. (TIF) [file pone.0225576.s002.tif]

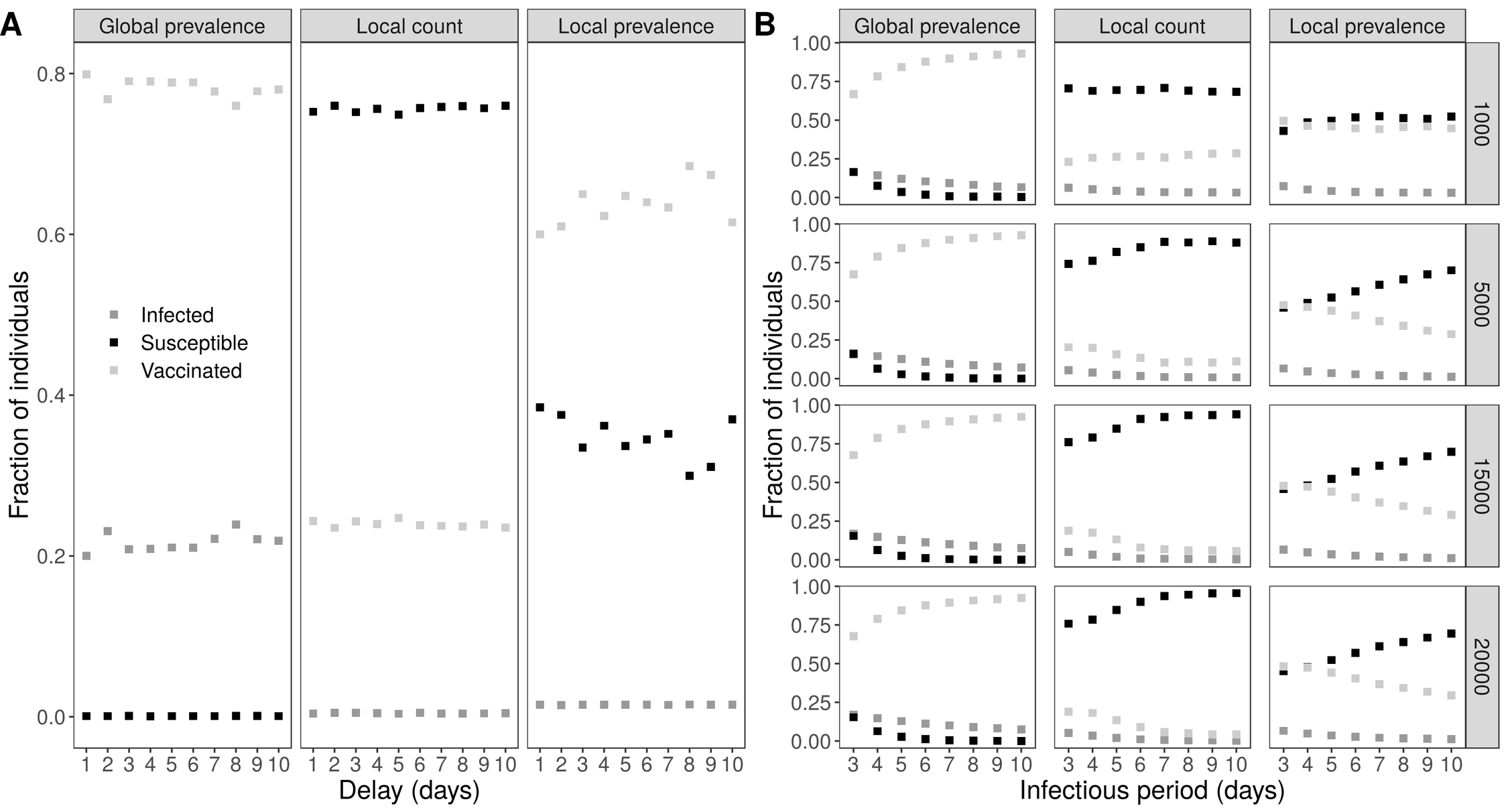

Supplement: S3 Fig — In both sets of graphs, the columns correspond to the three different strategies. Each point indicates a mean over 500 stochastic simulations that assume R0 = 5. (A) The x-axes indicate the delay between an individual deciding to vaccinate and becoming immune to infection. Each simulation assumes an exponential network with N = 10000 individuals and that 80% of vaccinated individuals become protected against infection (the remaining 20% remain fully susceptible). (B) The rows correspond to different network sizes (1000, 5000, 15000 and 20000). (TIF) [file pone.0225576.s003.tif]
